# Facilitating childbirth choice for positive postnatal mental health well-being among women: a Namibian case study

**DOI:** 10.3389/fgwh.2024.1277611

**Published:** 2024-03-15

**Authors:** S. Mlambo, H. J. Amukugo

**Affiliations:** ^1^ Welwitchia Health Training Centre, School of Nursing, Windhoek, Namibia; ^2^ University of Namibia School of Nursing and Public Health, Oshakati, Namibia

**Keywords:** midwives, mental health, postpartum, choices, positive childbirth

## Abstract

Childbirth affects women in a myriad of ways including feelings of tiredness, being overwhelmed, stressed, and having baby blues, and if not attended to, this can lead to postpartum depression, which is a mental condition that can have disastrous effects. Childbirth can affect the mental and physical status of a woman and having supportive midwives who guide women by giving adequate information is an issue of critical concern for a positive birth experience. The World Health Organisation (WHO) has emphasised the need to facilitate childbirth choices for women as a means of having a safe and memorable experience as the experience in childbirth affects the psychological status of a woman. Some women may experience worry and anxiety during labour and childbirth, which may be exacerbated by bias and a lack of childbirth choice facilitation during pregnancy. A negative childbirth experience may lead to negative psychological distress and postpartum depression, which will interfere with the bond between the mother, baby, and family. Midwives, thus, need to understand the emotional aspects that are attached to childbirth and be able to facilitate and support the emotional as well as the psychosocial needs of women under their care. However, there is a dearth of empirical evidence within the Namibian context that can provide direction and context-specific solutions to the present challenge. The current study followed a qualitative research design with an exploratory approach with one-on-one interviews with 10 midwives who were purposively selected. The midwives' experiences in this study depicted their zeal towards the issue at hand; however, what stood out were some barriers in the facilitation of childbirth choices (theme 1) as they expressed the shortages of staff, the timing of information, information sharing, and cultural influences as some of their experiences in facilitating childbirth. Furthermore, midwives shared a lack of provision for childbirth choice (theme 2) as the rights of women were not observed, and a lack of women-centred care despite protocols and guidelines being there, and yet they are not adhered to. In conclusion, midwives as primary caregivers actively need to provide unbiased childbirth information to achieve positive postpartum health. Initiating childbirth choices early in pregnancy gives women the time to weigh options and clearing of any misconceptions relating to childbirth types as well as reducing anxiety and fear of birth, which could lead to postpartum depression and by extension, the mental well-being of the women. Facilitating childbirth choices is critical in positive birth experiences and the management of childbirth as well as crafting guidelines and policy formulation that ensure a mentally healthy woman and society.

## Introduction and background

Facilitation among pregnant women includes the provision of unbiased comprehensive information, support, as well as guidance to expectant women and their families (1). The current study describes childbirth facilitation as the provision of evidence-based information to women and their families regarding the modes of childbirth for a positive birth experience. Childbirth facilitation affects the women directly after the whole process is over, and this can be both positive and negative, hence the facilitation aims at alleviating fear and concerns among pregnant women (1). If women have positive childbirth facilitation from skilled midwives, it means that they will be able to re-live the experiences with joy and appreciation, and this only happens if they are not affected emotionally and physically (2, 3).

Midwives taking care of women during pregnancy have a mandate to give women adequate professional information for informed decisions to be made, and this will help even in future pregnancies as adequate information and the action taken have a bearing on future choices and actions (4, 5). Health education by midwives during pregnancy helps the women to gain insightful understanding and therefore lead to them making informed decisions and resultantly reducing fear and anxiety, which if not handled well may develop into depression (1).

Depression is identified as the most prevalent mental health problem among pregnant women (6), which can be aggravated should facilitation during antenatal care is not fully provided. In addition, when childbirth is well-facilitated, women will believe that they are responsible for the outcome as they would have been involved all the way up to the end of the birthing process (7). Midwives' professional training encourages them to advocate for the physiological type of birthing as they believe in the natural way of giving birth (8), but not excluding the high-risk pregnancies that will need obstetric care, and some women end with caesarean childbirth out of necessity (8). Midwives acknowledge the presence of pathology in some women and they are trained to identify them, and when that happens midwives can refer women accordingly (9, 10). However, doctors being fearful of litigation, are more likely to recommend caesarean delivery for women be it nullipara or multipara (9, 11) and this will, as a result, inflate the unwarranted number of caesarean section childbirths. As earlier stated, not all women may birth physiologically, but efforts need to be made by health professionals to uphold beneficence to ensure that women are autonomous in their decisions and ensuring that the psychology of women is put into consideration (1).

Trevathan and Rosenberg (12) argue that the effects of surgical childbirth primarily affect the woman and infant, and this can also affect future generations. Some scholars (4, 12, 13) further argue that women are connected to a line of generations, and their history, whether positive or negative, will likely have an impact on the generations to come as human beings often identify with their predecessors. It is, therefore, imperative for midwives to have the background of the women under their care during facilitation to correct myths and give evidence-based advice (14).

Women understand labour and childbirth as an unknown territory and hence this makes it central for facilitation to be prioritised (15). An unknown territory steers up anxieties in women which can trigger stress and uncertainty which can lead to depression among pregnant women (1). Anxiety and depression during ANC can lead to postpartum depression that has lasting effects on the woman and thereby create an unpleasant postnatal period (16). Midwives in the public sector assume the choices of women; however, women need to be treated individually (17) and that facilitation begins at the first contact and this has to be an ongoing process from pregnancy and labour till birth (15).

Postpartum depression affects both the mental and physical health of the mother and the child (18). However, midwives are the primary caregivers of women during pregnancy, especially in the public sector, thereby facilitating birth choices for women during pregnancy for the positive mental health of all women in their care. The scope of practice (10) of the registered midwife in Namibia is elaborate in identifying the “patient” as the mother and the child (child here also refers to the unborn). With the above in mind, the midwife needs to be familiar with the scope of practice (10) to be able to identify the expectations in the profession and in caring for pregnant women.

The scope of practice (10) indicates that a midwife is required to render:

“…scientific application of the principles of midwifery … providing assistance and medical care … including scientifically based physical, psychological, social, educational, chemical and technological means… in the course of pregnancy …” (10)

Firstly, the midwife needs to base the practice on the principles of midwifery when offering facilitation to women in the ANC. Midwives base their principles on physiological birth and thus they promote vaginal birth by giving women enough evidence-based information about vaginal birth (8). However, midwives must include the following in the care of the woman: physical, psychological, social, educational, chemical and technological means applicable to health in the course of their pregnancy. Caring for a woman holistically means including all the above, that is, the physical aspect of the woman such as caring for the pregnancy with routine ANC check-ups to make sure that she is all right. In addition, there is the psychological dimension which includes the midwife getting to know the woman and asking about any previous experiences, anxieties and apprehensions that may be affecting the current pregnancy (19, 20).

A study conducted in South Africa showed evidence of misinformation as a woman was quoted saying “I was told after my baby was born that l could not breastfeed my baby ….I have HIV. But if I didn’t breastfeed everyone would know I had HIV. I did anyway…I saw the sangoma (traditional healer) who helped me” (21). The above clearly depicts a negative effect if facilitation is assumed and not actioned. There is, therefore, a need for healthcare providers to offer women evidence-based information that will enable them to make informed decisions. The above is supported by a study which was conducted in Pakistan which found that errors or loopholes in antenatal surveillance and an ineffective referral system are possible causes of an increase in caesarean section rates (22). There is surely a gap if the healthcare professional is unsure of the information they have to give to the woman in terms of childbirth choices, and this can lead to negative outcomes when the birth plans do not go according to expectations.

In addition to misinformed education, the healthcare system may present challenges or barriers that will affect the facilitation that needs to be provided to women and their families regarding childbirth choices as a way to avert possible psychological effects. According to Stirling et al. (17), these barriers include but are not limited to “incomplete, inaccurate, biased or unavailable patient education resources and lack of opportunity for pregnant patients to have meaningful conversations with their health care providers about their individual care” which is a major setback when women are being facilitated with childbirth choices. Stirling et al. (17) further proffer that women need to be equipped with all the information about the different available childbirth modes. The information ensures that the pros and cons of the modes of childbirth are given for a healthcare provider to be satisfied that the woman will make informed decisions based on evidence-based information.

Women are to be the central decision-makers in their care hence they need to be accorded that opportunity, and midwives have the mandate to advocate for women (19, 23, 24). In as much as midwives are advocates of physiological birth, there is a need for them to accord the responsibility of making decisions to women by giving them all the information they need to know. In doing so, the midwife develops a comprehensive plan that respects the woman's preferences and decisions (25).

*Pre-pregnancy and antenatal competencies* identify the midwife as the primary caregiver for women in the public healthcare sector of Namibia, and the midwives need to have sound knowledge of pregnancy for them to be able to give the women information that will help them in making decisions. The information to be given to the woman and family needs to be accurate and clear and meet individual needs. Midwives have the mandate to treat each woman individually, thus the promotion of woman-centred care as well as inclusivity and acknowledging the family as a possible support system (20, 26). The Sustainable Development Goals (SDGs) are clear on the health and well-being of all including the mental status.

The competency of midwives encourages the discussion of options, preferences and contingency plans with women and supports persons and respects their decisions. The competency dimension is clear in urging midwives to make childbirth facilitation for the woman and her family. The childbirth types are to be given as options to the woman when they come for ANC and the preferences of women must also be put into consideration (19, 26). In as much as the woman has preferences, unbiased information needs to be given in detail for the different childbirth types that are possibilities as this will curb uncertainties, apprehension and a sense of helplessness when the choice is not what happens during the process of giving birth (20). A woman is an autonomous being and she should thus be accorded such information sharing and not simply assuming that they know much about childbirth.

## Aim of the study

The study aimed to explore and describe the experiences of midwives' facilitation of childbirth choices among women for positive postnatal mental health as experienced by midwives in facilitating childbirth.

## Methodology

The study followed a qualitative design with an exploratory descriptive approach (27) which was deemed appropriate as it added the voices of the midwives regarding their experiences in facilitating childbirth choices. The population comprised midwives in the maternity department working in the ANC departments at the time of the study. A purposive sampling technique to identify participants who know the phenomenon under study was used. Information leaflets explaining the purpose of the study were given to midwives and the midwives returned the provided signed consent form to partake and thereafter some arrangements were then made to conduct the interviews.

The study included midwives with six months of experience or more for them to share their knowledge about the phenomenon under study. Midwives on leave and those not willing to take part in the study were excluded. The sample was determined by data saturation drawn from a total population of 26 midwives. Appointments with midwives were made during their free time and place of convenience so as not to interfere with their work. A total of 10 midwives were interviewed and they formed the sample for the study. Interviews were conducted in the English language as it is the official language of instruction and communication in the Namibian context. The researcher used a semi-structured interview guide which was formulated using the reviewed literature. The interview guide comprised two sections, one for demographic information and the second for the main question on experiences in the facilitation of childbirth choices. Data analysis followed Creswell's (28) six steps of data analysis, namely, preparation and organisation of data, exploration and coding of data, coding and building descriptions of the themes, presenting and reporting findings, interpreting the findings, and validating the accuracy of findings.

## Trustworthiness

The four principles of trustworthiness were applied in this study, namely, credibility, dependability, transferability, and confirmability. Credibility was ensured through one-on-one interviews with the midwives, identifying and noting down mannerisms as well as probing further to confirm the data. Prolonged engagement with the midwives was observed so that midwives' experiences of facilitating were explicit, and this was ensured by probing, and interviews lasted between forty-five and sixty minutes. The methodology of the study was described to the respondents in detail and the validation of the coded data with the co-author ensured dependability as a way to increase the chances of replicability of the study in other settings. As an objective in the main study, triangulation was done with other data sets as well as with literature to extend the confidence of the study results thus addressing confirmability (25, 29). In addition, verbatim extracts were used to ensure that the voices of midwives are heard with regard to their experiences in facilitating childbirth choices for positive postnatal mental health. Finally, the researchers ensured that data saturation was achieved as no new knowledge was being gathered from the respondents. Data saturation was allowed so as to address the transferability of the study to other contexts.

## Results

The demographic characteristics are depicted in Table 1 with the different variables that included pseudonyms, age, gender, and years of experience. The ages of the research participants ranged between 28 and 54 years, which gave the study a variety of participants and helped to provide views from people with different demographics and possible world-views. The sample included two accoucheurs and eight midwives, thus showing a low representation of males in the profession. However, this did not affect the data quality as the ratio of accoucheurs and midwives is a true reflection of the number of accoucheurs in the profession within the Namibian context (9). The varying degree of the years of experience from 3 years to 9 years was an added advantage as it brought out the views from the eyes of different but experienced participants hence giving in-depth information. Within the context under study, midwives are rotated in the maternity department hence the variation of the experiences and also not having many years in one department.

**Table 1:** Demographic characteristics of midwives.

Pseudonym	Age	Gender	Years of experience
M1	32	Female	3 years
M2	30	Female	3 years
M3	28	Female	6 years
M4	54	Female	5 years
M5	37	Female	3 years
M6	40	Male	9 years
M7	27	Male	3 years
M8	35	Female	2 years
M9	26	Female	4 years
M10	29	Female	3 years

### Emerging themes and sub-themes

Midwives were interviewed so as to understand how they facilitate women during ANC at public healthcare facilities. Two themes were identified, namely barriers to the facilitation of childbirth choices and lack of childbirth choices. Moreover, the main themes also had subthemes that were generated as follows: Theme One: Shortages of staff/workload, the timing of information giving, and information sharing during ANC; and Theme Two: Provision of rights of women regarding choice, lack of woman-centred care, enforcement of protocols and guidelines, and cultural influences of childbirth types as depicted in Table 2.

**Table 2:** Themes and subthemes of midwives’ experiences in facilitating childbirth-choices.

Theme	Sub-theme
Barriers to facilitation of childbirth-choices	• Shortages of staff/workload • Timing of information giving • Information sharing during ANC
Lack of childbirth-choices	• Provision of rights of women regarding choice • Lack of woman-centred care • Enforcement of protocols and guidelines • Cultural influences on childbirth types

### Barriers to the facilitation of childbirth choices (theme 1)

A barrier may be described as an obstacle that hinders the progress or the continuation of an event (30). In this study, barriers to facilitation were identified as the hindrances that midwives encounter knowingly or unknowingly when facilitating women with childbirth choices. The talk pertaining to choice should first identify the choice that the woman needs to make, and thereafter, the information given about the pros and cons of the choices that are available, and finally the support to be rendered to the woman in relation to the choice taken (31). In this study, the factors identified as hindering midwives from facilitating childbirth choices included but were not limited to the following: the shortage of staff/workload, the timing of information giving, and information sharing during ANC.

#### Shortage of staff/workload

Shortage of staff refers to the midwives who are working with the women in a unit not having a proper nurse-patient ratio to meet the standard care of the patients. Shortage of staff has a direct impact on the support and information that may affect maternal and neonatal well-being in the long run (32). In this study, midwives expressed that the healthcare professionals that are in the ANC departments did not tally with the women that they received each day for ANC visits. Midwives expressed having inadequate time with the pregnant women to give them individualised care which includes listening to all their anxieties. The above is supported by the following verbatim statements from the respondents:

M4: “*Maybe 10 to 15 min—it's not long and if they are many you also try to become faster so as not to waste time on one patient*”.

M5: “ *… when we see 150 patients—yah—and we are only 4 midwives at the moment so on average it's like plus or minus 40 patients per midwife per day and that is too much*”.

M3: “*… and also there is not a lot of staff in ANC—it makes it a bit hard to go really in-depth with everything pertaining the mode of delivery*”.

As illustrated above, midwives indicated that in as much as they may be willing to give health information to women, staff shortages make this difficult as they are always racing against time. This, however, does not justify the inadequate provision of information to women.

### Timing of information giving

According to the Inter Professional-Shared Decision Making model (23), timing is everything when it comes to shared decision-making, which is also vital in the facilitation of childbirth choices. In order for one to be able to make a decision, they need to be given a chance to reflect on the information and the choices available to them before they can commit to the final decision. The following verbatim statements cement the timing of the information given as reported by the midwives:

M3: “*In ANC we give health education to the women usually during their first ANC visit*”.

M2: “*My experience with women is that when they come to ANC for their first visit we give them health education … Yes, we give them information on the types of delivery mostly if they are about nine months or six months… It is good to give them information according to the trimester…*”

M6: “*Once you give them that education at an early stage they tend to forget*”*.*

M4: “*I think it's proper to start when it's in the third trimester when you start to ask them about that. Because when it's early and you talk to them about delivery they will not take you seriously because it (the pregnancy bump) is still small*”.

Midwives are the primary caregivers for pregnant women in the public healthcare sector in Namibia, hence they need to be equipped with knowledge for them to be able to facilitate childbirth choices. According to the Patient Charter of Namibia (33), women have a right to receive information that pertains to their health, and that will ensure a higher probability of positive birth experiences as supported by WHO (5).

M6: “*When they start ANC we tell them about delivery … and that the caesarean section is only done when there is a complication to save the baby or you. Just to give them information of how or why people get caesarean section*”.

M7: “*It is important for the patient to be aware of the advantages and disadvantages of the different modes because some patients are just misinformed by others that caesarean section is good. Some with information will refuse Caesar but if necessary the patient should be given information*”.

M6: “*…..they will end up saying they want C/S if you tell them information on that. If you keep repeating they feel it's useless*”

Midwives expressed that if women are given options in ANC, then they will choose the caesarean section. However, the available literature suggests otherwise, that if women are given adequate information, they will make the best-informed decision which ensures a positive birth experience and reduces anxiety in pregnancy and birth, thereby increasing positive postnatal experiences (16, 24).

### Lack of childbirth choices (theme 2)

Childbirth is a lifetime experience and each birth is different and hence needs to be noted and handled as such. Childbirth choices stem from the availability of information to the women for her to be able to make informed decisions (24, 34, 35). In this study, most midwives concurred that there is an assumption that if women were low risk then there was no urgency in providing information relating to the childbirth choices.

M6: “*Most primis—we told them that when it's caesarean they cut your flesh to open your uterus and this one it's not your own choice like in modern life when there is elective caesarean section. No l don’t want to feel the contraction*”.

M7: “*When we do our own examination we determine what type of delivery a woman is suitable to have. We do not ask them about what they want because when you ask them most patients will ask to go for caesarean. ………… they do not have the absolute right for the mode of delivery*”.

M4: “*No we are not discussing it—We are sending them especially the caesarean—We are sending them to the Dr to decide whether they are going to have a second one*”.

Midwives in this study refer women mostly to the Dr if there is any discussion of a caesarean section, meaning that they also forfeit their role as primary caregivers. Midwives need to ensure that they provide health education on the available birthing choices and this starts from the beginning of ANC as transparency during the process ensures patient satisfaction that is linked to a positive birth experience (16).

### Provision of rights for women regarding choice

Respectful maternity care (RMC) works through the many different networks to ensure that all the women under their care receive RMC which also ensures that the rights of women are not infringed. In the current study, some midwives highlighted the fact that women were given information regarding birth especially those who have had a previous caesarean section as indicated below:

M3: “*For example, someone who had a previous caesarean and you will have to counsel her and she will have to decide, discuss with the patient and tell them the disadvantages and advantages of vaginal birth and caesarean also, she will have to decide on the type of delivery they want to have*”.

M2: “*Yes we tell them about the advantages and disadvantages of delivering vaginally and caesarean if they are about to deliver*”.

M6: “*Women have the right to childbirth. If they want C/S we refer them to the Dr and the Dr also gives them health education*”.

M9: “*If they don’t have any risk we really do not discuss with them anything regarding caesarean otherwise they will get confused*”.

It is worth noting that some midwives gave information to women about the advantages and disadvantages of caesarean section in advocating for Vaginal Birth After Caesarean in this context, thus allowing women to be part of the process and thereby ensuring shared decision-making (16).

### Lack of woman-centred care

Woman-centred care refers to care that is individualised and that focuses on the woman independent of other women who present with a similar problem (20, 36–39) as a way to give information which is relevant to the particular woman. In this study, it was evident that midwives paid particular attention to giving health information to a group of women with different characteristics as evidenced by the verbatim extracts below:

M3: “*For women who do not have a high risk, what we do is that we do group health education in terms of just giving them everything*”.

M6: “*When they start ANC we tell them about delivery when they are in a group*”.

Patient-led care is a concern and this is being advocated for in the aspect of RMC. In another study, women in Canada expressed that it is important for them to lead in their own care (40) as this will make them feel respected. This notion was also alluded to by other scholars (20, 36, 37, 41) as the women feel involved and that they are leading in the facilitation of their childbirth by being given adequate information. Hence in this way, they will understand the different types of childbirth that are available and will be allowed to decide for themselves after having guidance from the professionals.

### Enforcement of protocols and guidelines

Protocols and guidelines are the blueprint for midwives and these need to be enforced and ensured to satisfy the client that is under their care as patient satisfaction improves the positive postnatal experience (16). Protocols may only be adopted and implemented if there is regular training to inform the users about the available protocols to ensure that they are understood (39, 42–44). Moreover, not only should the protocols and guidelines be taught to the health professionals, but it should be ensured that they are implemented by the consumers and in this case it is the women in ANC (39).

The standardisation of protocols and guidelines within a context should always be guided by evidence-based research that is context-specific (45), hence ensuring the quality of care for women. The assumption of choice among midwives that every woman “wants vaginal birth” should be a starting point in ensuring that all women have an equal share in the facilitation they receive from midwives. The following verbatim extracts substantiate the notion of going with the flow in public healthcare facilities.

M4: “*We just found it like that—We are only giving health talks without the mode of delivery—that is what I found there. If there is something to that I have not seen it*”.

M10: “*When I joined I also noticed that the talks don’t include mode of delivery—We ask them by following the pink card and in that way we will know how they will deliver*”.

M3: “*It is only on the first visit and it also depends on who is giving it but the mode of delivery really is not part of it*”.

The available protocols in Namibia are the Patient Charter (33) and the Scope of Practice (10), which reiterate the importance of the information that is given to patients for them to have informed choices but these are not discipline specific to childbirth choices.

### Cultural influences on childbirth-choices

Culture is the way of life that a certain group of people adopts and lives by. Childbirth choices are no exception to the cultural influences that come with them among women when it comes to giving birth. In this study, midwives attested to the fact that there are instances when the woman needs a caesarean section birth but due to the society they come from, this becomes difficult as is evidenced by the verbatim extracts below:

M4: “*….. maybe to be cut for the fourth time or what—maybe they can have sterilisation or what—its them to choose. Some of them it's like no I am going to talk to my husband or what, and then they will come back with what the partner says*”.

M9: “*Some women when you tell them that they are going for a caesarean they will tell you that they first want to talk to their relatives or husbands to know if it's the right way*”.

Preis et al. (46) proffer that the beliefs of women need to be put into consideration when birth is concerned. Involving women and giving them the time to process the information about birth may help them to understand and have a positive childbirth experience (47).

## Discussion

Information sharing in ANC plays a crucial role in enabling positive mental health. In the present study, it was found that there was minimal one-on-one engagement to give information in-depth. Fear of birth and not being heard were identified as factors that lead to postpartum depression among women (48) as women feel that they have lost control of their health. It is, therefore, important to prepare women and their families psychologically for any events that may occur during pregnancy as a contingency plan and a mark of preparedness (10, 17). Contrary to the mantra of some midwives in this study that if women are informed about caesarean section as an option then they will opt for that mode of birth, actually giving information and hearing about the preferences of women makes them feel involved and heard, and this reduces the incidences of postpartum depression and birth dissatisfaction (1, 48).

The enforcement of protocols and guidelines in the current study was found to be lacking to a greater extent, which was also a result of staff shortages for the midwives to give individualised care to the women during ANC. The general competencies of a midwife (8) allude to the midwife being aware of the scope of practice and the laws (10) that govern their practice and being accountable as a professional. In addition, midwives are aware of the relationships that they have with women and they should as such ensure that practice is evidence-based. Midwives are expected to uphold the trust of the public, which means that women and society look up to the midwives as the epitome of knowledge and that they are there to help the women when they are in ANC (8, 10).

In the current study, midwives assumed that if women were given a choice, they would choose caesarean section as the mode of delivery. However, midwives are there to educate women about their rights in sexual reproductive health hence the principle of autonomy in decision-making by the women. Autonomy to choose in childbirth is a right that all women deserve but women need to be informed, with unbiased information timeously so as to make informed decisions about the mode of childbirth they believe is best for them (34). The opportunity to partake in decision-making regarding childbirth leads to women who exhibit positive mental well-being in the postnatal period thereby reducing the incidence of negative postpartum health among women.

Midwives expressed not having enough time with pregnant women during antenatal visits and this concurs with the study findings by Hastings-Tolsma et al. (21). Some of the women expressed that no information was given to them during the visits hence it was either what the woman knew superficially or what the healthcare provider advised (21). According to the informed decision-making model by Stirling et al. (17), pregnancy with no complication is carried for nine months hence this is ample time for healthcare providers to facilitate sound and informed decision-making for women under their care. Furthermore, Stirling et al. (17), affirm that most women require a longer period for them to be able to make a rational decision hence it is logical to say that the facilitation of childbirth choices should be initiated at the beginning of antenatal care. Initiating the facilitation of childbirth choices early gives the woman time to weigh the options and for the healthcare provider to clear any misconceptions relating to childbirth modes (21, 22, 24).

## Conclusion

Midwives in this study alluded to experiencing less time with women during the antenatal period leading to inadequate facilitation of childbirth choices including giving and identifying information needs for the women regarding childbirth. A positive birth experience stems from the satisfaction of the process from pregnancy to birth and positively affects the mental status of the woman. Baby blues postpartum may be avoided by woman-centred facilitation during pregnancy and resultantly reduce the incidence of postpartum depression among women in the postpartum period. A gap was identified in this study with regards to midwives understanding previous birth experiences preferences regarding mode of birth as a mitigating factor for negative postpartum health. Postpartum wellness of women would be attained if they are involved in the shared decision-making of their health. Midwives must facilitate childbirth choices taking into account that pregnancy and childbirth experiences are significant in contributing to positive postnatal mental health among women.

## Data Availability

The original contributions presented in the study are included in the article/supplementary material, further inquiries can be directed to the corresponding author.

## References

[1] Khan B Hameed W Avan BI. Psychosocial support during childbirth: development and adaptation of WHO’s mental health gap action program (mhGAP) for maternity care settings. PLoS ONE. (2023) 18(5):e0285209. 10.1371/journal.pone0285209 37216373 PMC10202270

[2] International Federation of Gynecology and Obstetrics (FIGO). Mother-baby-friendly birthing facilities. Int J Gynecol Obstet. (2014) 128(1):95–9. 10.1016/j.ijgo.2014.10.013 25467915

[3] World Health Organisation (WHO). Intrapartum Care for a Positive Childbirth Experience. Geneva: World Health Organisation (2018). 30070803

[4] Grigg C Tracy SK Daellenbach R Kensington M Schmied V. An exploration of influences on women’s birthplace decision making in New Zealand: a mixed methods prospective cohort within the evaluating maternity units study. BMC Pregnancy Childbirth. (2014) 14(210):1–12. 10.1186/1471-2393-14-210 24951093 PMC4076764

[5] World Health Organisation. Catalogue of Resources to Support Health Services. Geneva: Delivery Transformations (2016). Available online at: https://www.euro.who.int/_data/assets/pdf_file/0010/317791

[6] Bedaso A Adams J Peng W Sibbritt D. The relationship between social support and mental health problems during pregnancy: a systematic review and meta analysis. Reprod Health. (2021) 18(1):162. 10.1186/s12978-021-01209-5 34321040 PMC8320195

[7] Konheim-Kalkstein Y Kirk CP Berish K Galotti KM. Owning the birth experience: what factors influence women’s vaginal birth after caesarean decision. J Reprod Infant Psychol. (2017) 35(4):410–22. 10.1080/02646838.2017.1320365 29517376

[8] International Confederation of Midwives. (2018). *Advocacy toolkit*. Available online at: www.internationalmidwives.org

[9] Mlambo S. (2018). *Midwives’ views on delivery method decision-making in private sector labour wards of Namibia*. Masters thesis. University of Stellenbosch, Cape Town, South Africa. Available online at: www.scholar@sun.ac.za

[10] Republic of Namibia. Nursing Act 8 of 2004. Windhoek: Government Printer (2004).

[11] Cheng Y Li X Lou C Sonestein FL Kalamar A Jeieebhov S The association between social support and mental health among vulnerable adolescents in five cities: findings from the study of the well-being of adolescents in vulnerable environments. J Adolesc Health. (2014) 55(6 Suppl):S31–S38. 10.1016/j.jadohealth.2014.08.020 25454000

[12] Trevathan WR Rosenberg KR. Caesarean section. Evol Med Public Health. (2014) 2014:164. 10.1093/emph/eou031 PMC4277241 25504678

[13] Dehingia N Dixit A Atmavilas Y Chandurkar D Singh K Silverman J Unintended pregnancy and maternal health complications: cross-sectional analysis of data from rural Uttar Pradesh, India. BMC Pregnancy Childbirth. (2020) 20:188. 10.1186/s12884-020-2848-8 32228511 PMC7106893

[14] Lee S Ayers S Holden D. How women with high-risk pregnancies perceive interactions with healthcare professionals when discussing the place of birth: a qualitative study. Midwifery. (2016) 38:42–8. 10.1016/j.midw.2016.03.009 27046264

[15] Borelli SE Walsh D Spiby H. First-time mothers ‘expectations of the unknown territory of childbirth: uncertainties, coping strategies and ‘going with the flow’. Midwifery. (2018) 63:39–45. 10.1016/j.midw.2018.04.022 29778717

[16] Arrebola RN Mahia LP Lopez SB Castineira NL Pillado TS Sonia Pertega Diaz S. Women’s satisfaction with childbirth and postpartum care and associated variables. Rev Da Escola De Enferm Da USP. (2021) 55:e03720. 10.1590/S1980-220X2020006603720 34133650

[17] Stirling D Vanbesien J McDougall R. Informed Decision-Making for Labour and Birth. 2nd ed. Ontario: Ontario Public Health Association (2018). Available online at: www.opha.on.ca

[18] Liu X Wang S Wang G. Prevalence and risk factors of postpartum depression in women: a systematic review and meta-analysis. J Clin Nurs. (2021) 31:2665–77. 10.1111/jocn.16121 34750904

[19] Mlambo S Morgan-Cramer J Young C. Women’s decision-making on birthing choices in the private sector of Namibia: Midwives’ perspectives. Afr J Nurs Midwifery. (2020) 22(1):1–13. 10.25159/2520-5293/6431

[20] Benyamini Y Molcho ML Dan U Gozlan M Preis H. Women’s attitudes towards the medicalization of childbirth and their associations with planned and actual modes of birth. Women Birth. (2017) 30:424–30. 10.1016/j.wombi.2017.03.007 28434672

[21] Hastings-Tolsma M Notle AGW Temane A. Birth stories from South Africa: voices unheard. Women Birth. (2018) 31:e42–50. 10.1016/j.wombi.2017.06.015 28711397

[22] Ahmad M Ahmad MQ Sohail CS Abdullah M Sohail T Jamal A Social and demographic determinants of mode of delivery among pregnant women visiting gynaecology department of jinnah hospital lahore, Pakistan. J Fam Med Community Health. (2018) 5(4):11–56. 10.47739/2379-0547/1156

[23] Legare F Stacey D, IP Team. (2010). *Interprofessional shared decision-making model*. Available online at: www.ohri.ca/decisonaid

[24] Loke AY Davies L Li S. Factors influencing the decision that women make on their mode of delivery: the health belief model. BmC Health Serv Res. (2015) 15:274. 10.1186/s12913-015-0931-z 26188472 PMC4506759

[25] Mlambo S. (2022). *A model for midwives towards the facilitation of childbirth choices among women in selected public healthcare facilities in Namibia*. Doctoral thesis. University of Namibia, Namibia. Available online at: http://hdl.handle.net/11070/3581

[26] Feeley C Thomson G Downe S. Understanding how midwives employed by the national health service facilitate women’s alternative birthing choices: findings from a feminist pragmatist study. PLoS One. (2020) 15(11):e0242508. 10.1371/journal.pone.0242508 33216777 PMC7678977

[27] Grove SK Gray JR. Understanding Nursing Research: Building an Evidence-Based Practice. Philadelphia: Saunders (2018).

[28] Creswell JW Creswell JD. Research Design: Qualitative, Quantitative and Mixed Methods Approaches. Los Angeles: SAGE Publications. 5th ed. (2018).

[29] Mlambo S Amukugo HJ. Childbirth-choice facilitation experiences among women in selected public healthcare facilities in Namibia. Midwifery. (2023) 126:103835. 10.1016/j.midw.2023.103835 37804668

[30] Oxford Online Dictionary. (n.d.). Available online at: https://www.oxfordlearnersdictionaries.com/ (accessed July 12, 2023).

[31] Nieuwenhuijze MJ Low LK. Facilitating women’s choice in maternity care. J Clin Ethicaesarean Sec. (2013) 24(3):276–82. 10.1086/JCE201324311 24282854

[32] Turner L Culliford D Ball J Kiston-Reynolds E Griffiths P. The association between midwifery staffing levels and the experiences of mothers on postnatal wards: cross-sectional analysis of routine data. Women Birth. (2022) 35(6):e583–9. 10.1016/j.wombi.2022.02.005 35183474

[33] Ministry of Health and Social Services. Patient Charter. Windhoek, Namibia (nd). Available online at: https://mhss.gov.na/documents/146502/2041604/MOHSS+PATIENT+CHARTER+%281%29.pdf/16502fb1-7242-fa4e-3f48-8707a96afe59?t=1685540925569 (accessed May 15, 2023).

[34] Attanasio LB Kozhimannil KB Kjerulff KH. Factors influencing women’s perceptions of shared decision-making during labour and delivery: results from a large-scale cohort study of first childbirth. Patient Educ Couns. (2018) 101(6):1130–6. 10.1016/j.pec.2018.01.002 29339041 PMC5977392

[35] Davis D Homer CS Clack D Turkman S. Choosing vaginal birth after caesarean section: motivating factors. Midwifery. (2020) 88:1–6. 10.1016/j.midw.2020.102766 32526606

[36] Boz I Teskereci G Akman G. How did you choose a mode of birth? Experiences of nulliparous women from Turkey. Women Birth. (2016) 29:359–67. 10.1016/j.wombi.2016.01.005 26846560

[37] Bringedal H Aune I. Able to choose? Women’s thoughts and experiences regarding informed choices during birth. Midwifery. (2019) 77:123–9. 10.1016/j.midw.2019.07.007 31323487

[38] Chen M McKellar L Pincombe J. Influences on vaginal birth after caesarean section: qualitative study of Taiwanese women. Women Birth. (2017) 30:e132–9. 10.1016/j.wombi.2016.10.009 27818106

[39] Coates D Thirukumar P Henry A. Making shared decisions in relation to planned caesarean sections: what are we up to? Patient Educ Couns. (2020) 103:1176–90. 10.1016/j.pec.2019.12.001 31836248

[40] Vedam S Stoll K McRae DN Korchinski M Velasquez R Wang J Patient-led decision making: measuring autonomy and respect in Canadian maternity care. Patient Educ Couns. (2019) 102:586–94. 10.1016/j.pec.2018.10.023 30448044

[41] Afulani PA Phillips B Aborigo RA Moyer CA. Person-centred maternity care in low-income and middle-income countries: analysis of data from Kenya, Ghana and India. Lancet. (2019) 7(1):e96–109. 10.1016/S2214-109X(18)30403-0 PMC6293963 30554766

[42] Feeley C Thomson G. Tension and conflicts in ‘choice’: ‘Women’s’ experiences of free birthing in the UK. Midwifery. (2016) 41:16–21. 10.1016/j.midw.2016.07.014 27498184

[43] Hinton L Dumelow C Rowe R Hallowell J. Birthplace choices: what are the information needs of women when choosing where to give birth in England? A qualitative study using online and face-to-face focus groups. BMC Pregnancy Childbirth. (2018) 18:12. 10.1186/s12884-017-1601-4 29310599 PMC5759241

[44] Newnham E McKellar L Pincombe J. ‘It’s your body, but…’ mixed messages in childbirth education: findings from hospital ethnography. Midwifery. (2017) 55:53–9. 10.1016/j.midw.2017.09.003 28942214

[45] Diamond-Brown L. It can be challenging, it can be scary, it can be gratifying: Obstetricians’ narratives of negotiating patient choice, clinical experience, and standards of care in decision-making. Soc Sci Med. (2018) 205:48–54. 10.1016/j.sectioncimed.2018.04.002 29635190

[46] Preis H Eisner M Chen R Benyamini Y. First-time mothers' birth beliefs, preferences, and actual birth: A longitudinal observation study. *Women Birth.* (2019) 32:e110–7. 10.1016/j.wombi.2018.04.019 29753684

[47] Nascimento RRP Arantes SL de Souza EDC Contrera L Sales APA. Choice of type of delivery: factors reported by puerperal women. Rev Gaucha Enferm. (2015) 36(spe):119–26. 10.1590/1983-1447.2015.esp.56496 27057710

[48] Henriksen L Grimsrud E Schei B Lukasse M Group BS. Factors related to a negative birth experience—a mixed methods study. Midwifery. (2017) 51:33–9. 10.1016/j.midw.2017.05.004 28528179

